# Serum soluble interleukin-2 receptor levels for screening for malignant lymphomas and differential diagnosis from other conditions

**DOI:** 10.3892/mco.2019.1922

**Published:** 2019-09-11

**Authors:** Jun Murakami, Kotaro Arita, Akinori Wada, Hiroshi Mihara, Hideki Origasa, Mika Kigawa, Ichiro Yasuda, Tsutomu Sato

**Affiliations:** 1 Department of Gastroenterology and Hematology, University of Toyama, Graduate School of Medicine and Pharmaceutical Sciences, Toyama 930-0194, Japan; 2 Division of Biostatistics and Clinical Epidemiology, University of Toyama, Graduate School of Medicine, Toyama 930-0194, Japan; 3 Faculty of Health and Welfare, Kanagawa University of Human Services, Yokosuka, Kanagawa 238-8522, Japan

**Keywords:** serum soluble interleukin-2 receptor, malignant lymphoma, screening, tumor marker, diagnostic value

## Abstract

The serum soluble interleukin 2 receptor (sIL2R) level is elevated in patients with most types of lymphoid neoplasms, and is also elevated in patients with solid tumors or reactive conditions, such as severe inflammation. To evaluate the diagnostic significance of sIL2R levels for the screening and differential diagnosis of lymphomas, data from 248 consecutive adult patients with suspected lymphoma were retrospectively analyzed in order to determine its diagnostic characteristics and the clinical parameters that affect diagnosis. In 133 patients with aggressive or indolent lymphomas or related neoplasms, the sIL2R level was higher (median: 920 U/ml, standard deviation: 7,312 U/ml) compared with that of 115 patients with other diagnoses (median: 520 U/ml, standard deviation: 727 U/ml), including solid tumors, infection, inflammation, and others. When the cutoff value of sIL2R was 1,104 U/ml, the specificity was 80%, at which point lymphoma was suspected. When the threshold levels were increased from 1,500 to 2,000 U/ml, the specificity increased from 87 to 93%, with the positive likelihood ratio increasing from 2.99 to 4.97, strongly suggesting the diagnosis of lymphoma. The receiver operating characteristic curve for prediction of lymphoma by sIL2R revealed that the area under the curve was 0.695. The curve was nearest to the left corner of the plot when the threshold was 1,946 U/ml; at this point, the sensitivity, specificity and positive likelihood ratio were 35%, 93% and 5.06, respectively. Multivariate analysis demonstrated that an age >46 years and lactate dehydrogenase level >173 U/l appeared to increase the risk of malignant lymphoma diagnosis. Although sIL2R appears to be a less specific marker for the screening of lymphomas, its detection at higher levels strongly suggests the diagnosis of lymphomas. Therefore, sIL2R may be more useful compared with any other parameter for lymphoma diagnosis, provided other false-positive conditions are taken into consideration.

## Introduction

Interleukin 2 (IL-2), which has been identified in activated T cells, stimulates an immune response on target cells via a high affinity receptor composed of α, β and γ subunits. The interaction between IL-2 and its receptors on target cells plays a pivotal role in activating and maintaining immune responses. The soluble form of the IL2R α subunit (CD25) appears to be released into the serum from the membranes of activated lymphocytes after shedding by proteolytic cleavage. Soluble IL-2 receptor α (sIL2R) is detected in the serum of healthy individuals and increases in association with various types of inflammation or neoplasms (1–3). However, the mechanism and functional significance of elevated sIL2R levels for each pathological condition remain unclear.

Serum sIL2R levels have been found to be elevated in most types of hematolymphoid neoplasms, including Hodgkin's lymphomas, non-Hodgkin lymphomas, acute lymphoblastic leukemia (ALL), chronic lymphocytic leukemia (CLL), multiple myeloma, and others (3). Moreover, the highest levels of sIL2R have been reported in adult T-cell lymphoma/leukemia (ATLL) (4) and hairy cell leukemia (5). In patients with hematolymphoid neoplasms, most of the serum sIL2R is derived from the neoplastic cells themselves, directly reflecting the tumor burden and disease activity; therefore, it may be considered as a true tumor marker. In certain B-cell lymphomas, proteinases derived from tumor-associated macrophages in the tumor microenvironment also appear to play an important role in producing sIL2R (6). In several non-lymphoid solid tumors, serum sIL2R levels are significantly higher compared with those in healthy individuals, although this increase is not as notable as that observed in hematolymphoid neoplasms (1,7,8). In non-lymphoid solid tumors, increased serum sIL2R levels may originate from tumor cells, as well as from activated lymphoid cells, circulating mononuclear cells, or tumor-infiltrating lymphocytes (3). Furthermore, elevated sIL2R levels have been detected in a growing number of pathological conditions, including infections, autoimmune or other inflammatory diseases, allograft rejection, graft-vs.-host disease after allogeneic hematopoietic stem cell transplantation, and hemophagocytic lymphohistiocytosis (3).

Although the mechanisms and clinical significance of elevated sIL2R levels are unclear, the clinical usefulness of sIL2R has been evaluated for the diagnosis, staging, prognosis and post-treatment monitoring in lymphomas or other diseases (3). However, the availability of data on the significance of sIL2R levels in the differential diagnosis of lymphoma from other diseases in the clinical setting is limited (1,7,8). In the present study, patients with suspected lymphoma were retrospectively analyzed and the sIL2R levels were measured, the final diagnoses were compared, and then the diagnostic characteristics and clinical parameters that affect the diagnostic value were evaluated.

## Patients and methods

### 

#### Ethics approval

The present study was approved by the Ethics Committee of the University of Toyama. The use of an opt-out method enabled refusal to participate in disclosure documents.

#### Patient population

A total of 248 consecutive adult patients (aged ≥18 years) who had suspected malignant lymphoma and had their serum sIL2R levels measured by attending physicians in Toyama University Hospital between January 2004 and December 2007 were retrospectively analyzed.

#### Data collection

Data on clinical parameters, including age, sex, white blood cell (WBC) count, C-reactive protein (CRP) levels (normal range: 0.00–0.14 mg/dl), serum lactate dehydrogenase (LDH) levels (normal range: 124–222 U/l) and serum sIL2R levels, were extracted from the medical record database. Clinical symptoms at presentation, initial differential diagnosis and final diagnosis were reviewed in the medical records for each patient. Standard histological diagnosis of lymphoma by hematopathologists was based on the World Health Organization 2008 classification.

#### sIL2R measurement

The serum sIL2R levels were measured with a sandwich-enzyme immunoassay (IL-2R test; BML; cat. no. 221ADAMX00007000). The normal range was defined as 122–496 U/ml following the manufacturer's information.

#### Statistical analyses

The sensitivity (Sn) and specificity (Sp) of the sIL2R levels for the diagnosis of lymphoma and other lymphoid neoplasms were evaluated, and the positive and negative predictive values were also determined. A receiver operating characteristic (ROC) curve was used to determine the diagnostic accuracy and cutoff value of sIL2R. Differences between the two groups were evaluated using the Mann-Whitney U test. Comparisons among multiple groups were made using the Kruskal-Wallis test, and post hoc group comparisons were performed with the Steel-Dwass test. A multivariate logistic regression model was used to determine risk factors for lymphomas. All data were considered statistically significant if the P-values were <0.05. The analyses were performed using EZR version 2.4-0 (Saitama Medical Center, Jichi Medical University), which is a graphical user interface for R (The R Foundation for Statistical Computing) (9).

## Results

### 

#### Patient characteristics

A total of 248 patients with lymphoma suspected by attending physicians based on clinical, laboratory or imaging findings were included in the present study. Lymphoma was suspected due to varying reasons. The common presentations and differential diagnoses for lymphomas included solid tumors with uncommon presentation, inflammatory symptoms, liver dysfunction, neurological symptoms of unexplained etiology, or even unexplained complaints. The final diagnosis of lymphoma subtype or differential diagnoses were established. The patient characteristics are summarized in Table I. Of the 248 patients, 97 were diagnosed with aggressive lymphomas, 23 with indolent lymphomas, 8 with plasma cell disorders including plasma cell myeloma, and 5 with CLL. The remaining 115 patients were diagnosed with neoplasms other than lymphoid. The most common diagnosis was non-lymphoid solid tumor (n=50), followed by infection (n=32), non-infectious inflammatory disorders (autoimmune diseases or allergy; n=18) and miscellaneous (n=15).

The histological subtypes of aggressive or indolent lymphomas and other lymphoid neoplasms are listed in Table II. Among patients diagnosed with non-lymphoid solid neoplasms, the most common types of tumors were of gastrointestinal or urogenital origin, followed by primary unknown carcinomas, non-epithelial tumors, abdominal lymph node enlargement, and splenic or bone tumors without histological confirmation.

#### Comparison of each parameter in patients with lymphoid tumors or other diagnoses

The sIL2R levels and other parameters were compared between the two groups: Malignant lymphomas, including CLL and plasma cell disorders (ML group) and other diagnoses (other group) (Table III). The median age of the patients in the ML group was significantly higher compared with that in the other group (67 years vs. 60 years, respectively; P=0.02). The median WBC count in the ML group was similar to that of the other group (4,570/*µ*l vs. 4,680/*µ*l). The median serum CRP level in the ML group was somewhat higher compared with that of the other group, but the difference was not statistically significant (0.4 mg/dl vs. 0.2 mg/dl, respectively; P=0.066). The median LDH level in the ML group was significantly higher compared with that in the other group (217 U/l vs. 182 U/l, respectively; P<0.01). The median serum sIL2R level in the ML group was significantly higher compared with that in the other group (920 U/ml vs. 520 U/ml, respectively; P<0.001). Similar results were observed if patients with CLL and plasma cell disorders were extracted from the lymphoma group (data not shown).

#### sIL2R levels in each category and subcategory

**Figure 1.**
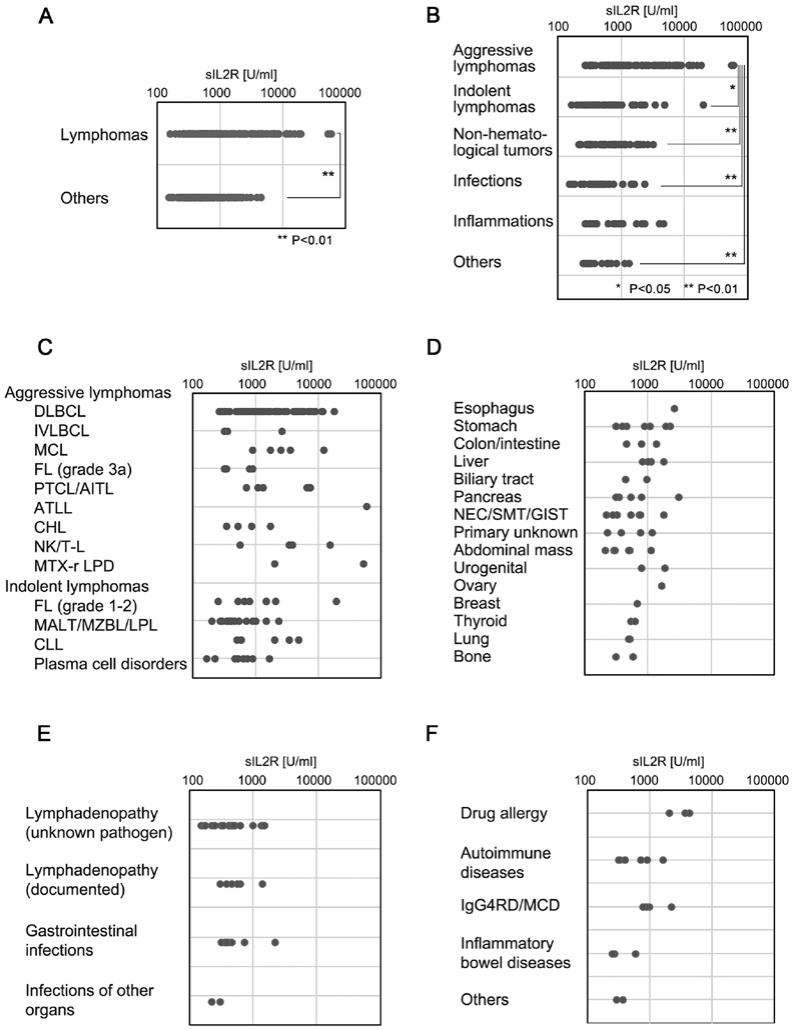
sIL2R levels in each category and subcategory. Dot plot of serum sIL2R levels in each diagnosis category. (A) Comparison of the serum sIL2R levels in the lymphoma group and the non-lymphoid group. (B) Serum sIL2R levels for each disease category, including aggressive or indolent types of lymphomas, CLL, plasma cell disorders including plasma cell myeloma, infection, non-infectious inflammation, and others. (C) Serum sIL2R levels of each of the detailed subtypes of aggressive lymphomas, indolent lymphomas, CLL and plasma cell disorders, including plasma cell myeloma. (D) Serum sIL2R levels in various non-lymphoid tumors. (E) Serum sIL2R levels in various infectious diseases. (F) Serum sIL2R levels in various inflammatory diseases. sIL2R, soluble interleukin-2 receptor; CLL, chronic lymphocytic leukemia; DLBCL, diffuse large B-cell lymphoma; IVLBCL, intravascular diffuse large B-cell lymphoma; MCL, mantle cell lymphoma; FL, follicular lymphoma; AITL, angioimmunoblastic T-cell lymphoma; PTCL, peripheral T-cell lymphoma; ATLL, adult T-cell leukemia/lymphoma; CHL, classical Hodgkin's lymphoma; NK/T-L, natural killer/T-cell lymphoma; MTX-r LPD, methotrexate-related lymphoproliferative disorders; MALT, extranodal mucosa-associated lymphoid tissue type lymphoma; MZBL, nodal marginal zone B-cell lymphoma; LPL, lympho-plasmacytic lymphoma; NEC, neuroendocrine carcinoma; SMT, submucosal tumor; GIST, gastrointestinal stromal tumor; IgG4-RD, IgG4-related disease; MCD, multicentric Castleman's disease.

The comparison between serum sIL2R levels in the ML and the other groups is presented in Fig. 1A. The serum sIL2R levels in each of the disease categories are shown in Fig. 1B. Although aggressive lymphomas were associated with the highest sIL2R levels, indolent types of lymphomas appeared to have sIL2R levels similar to those of other disease categories, including non-hematological tumors and infection, among others.

The ML group was subcategorized into aggressive or indolent histological types, CLL and plasma cell disorders, including plasma cell myeloma. Aggressive lymphomas exhibited the highest sIL2R levels, followed by indolent lymphomas, CLL and plasma cell disorders (Fig. 1C). One patient with ATLL (acute type) had the highest levels of sIL2R (58,089 U/ml). The sIL2R level in patients with aggressive T/NK lymphomas except ATLL (median, 5,080 U/ml; range, 556–15,087 U/ml) was significantly higher (P<0.002) compared with that in patients with aggressive B-cell lymphomas (median, 1,046 U/ml; range, 257–17,709 U/ml).

The sIL2R levels in various non-lymphoid solid tumors are shown in Fig. 1D. Of note, 2 of 3 patients with extremely high sIL2R levels (>2,000 U/ml) had advanced tumors with splenic involvement. The sIL2R levels in various infectious diseases are shown in Fig. 1E. Mild or moderate increases in sIL2R levels were observed. The sIL2R levels in various inflammatory diseases are shown in Fig. 1F. Of note, 3 of 4 patients with sIL2R levels >2,000 U/ml had a drug allergy or another IgG4-related disease (Mikulicz disease).

#### Chronological increase of sIL2R levels in relation to disease progression

In our cohort, 13 patients with diffuse large B-cell lymphoma had their sIL2R levels re-measured prior to treatment (interval range, 19–168 days; median, 27 days). The evaluation of sIL2R levels (median, 2,685 U/ml) revealed that they had increased from the initial measurement (median, 1,156 U/ml), consistently with disease progression (P=0.0198).

#### Diagnostic value of serum sIL2R level for the diagnosis of malignant lymphoma

Patients in the ML and other groups were divided into sIL2R increase-positive and -negative populations by adjusting the cutoff value of the sIL2R level 500–5,000 U/ml by every 500 or 1,000 U/ml (Table IV). The Sn decreased from 47.4 to 15% by incrementing the point of the cutoff value, whereas the Sp increased from 76.5 to 100%. When the Sp was 80%, the threshold level of sIL2R was 1,104 U/ml, at which point ML was suspected. When the cutoff value was increased up to 1,500 U/ml, the Sp, odds ratio, and positive likelihood ratio (LR+) were elevated to 87%, 4.28, and 2.997, respectively. Even when the cutoff value was adjusted to 2,000 U/ml, the Sp, odds ratio and LR+ were elevated to 93.2%, 17.07 and 5.058, respectively.

**Figure 2.**
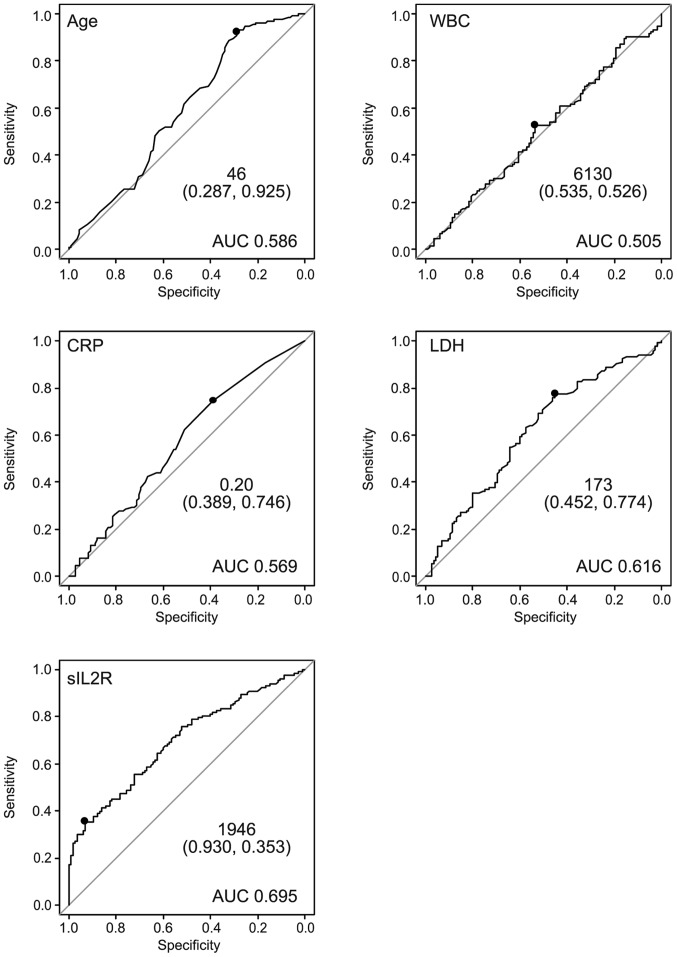
The ROC curve for prediction of lymphoma by several clinical parameters. The ROC curve for prediction of lymphoma by several clinical parameters (age, WBC count, CRP, LDH, sIL2R). WBC and CRP did not appear to contribute to the diagnosis of ML. By contrast, age, LDH, and sIL2R appeared to be of diagnostic value for the presence of ML. ROC, receiver operating characteristic; WBC, white blood cell; CRP, C-reactive protein; LDH, lactate dehydrogenase; sIL2R, soluble interleukin-2 receptor; AUC, area under the ROC curve; ML, malignant lymphoma.

The ROC curve for prediction of lymphoma by sIL2R is shown in Fig. 2. The area under the curve (AUC) was 0.695. The curve was nearest to the left corner of the plot when the threshold was 1,946 U/ml, at which point the Sn and Sp were 34.6 and 93.2%, respectively. The positive predictive value (PPV) and negative predictive value (NPV) at this threshold were 85.2 and 55.6%, respectively. This threshold was considered appropriate, as the Sp declined rapidly when a threshold of <1,946 was applied. Age and LDH levels appeared to contribute to the diagnosis of ML. By contrast, WBC and CRP did not appear to be predictive of the presence of ML. The ROC curve was nearest to the left corner of the plot when the thresholds were as follows: Age 46 years, LDH 173 U/l, and sIL2R 1,946 U/ml (Fig. 2).

#### Risk factors in the diagnosis of malignant lymphoma by multivariate analysis

**Figure 3.**
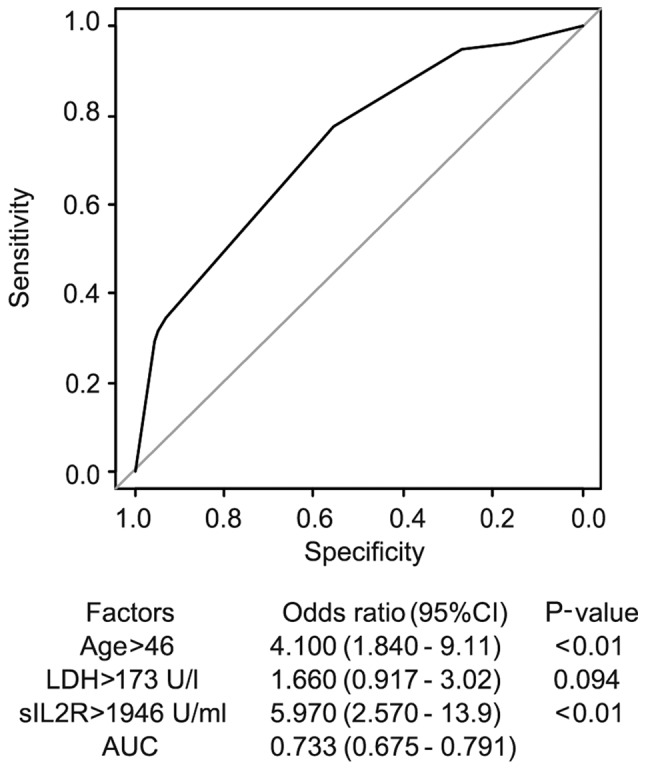
Multivariate logistic regression model: Risk factors for malignant lymphoma. The AUC of the sIL2R level was elevated to 0.733 by including participants aged >46 years and LDH levels of >173 U/l to the sIL2R cut-off value at 1,946 U/ml. AUC, area under the receiver operating characteristic curve; sIL2R, soluble interleukin-2 receptor; LDH, lactate dehydrogenase.

The multivariate analysis of the serum sIL2R level and several factors (age, sex, presence of fever, WBC count, LDH and sIL2R) was performed using the multivariate logistic regression model. The AUC was 0.695 for sIL2R alone, and increased to 0.733 by including age >46 years an LDH >173 U/l with the sIL2R cutoff value at 1,946 U/ml (Fig. 3). The adjusted odds ratio of the sIL2R level was 5.97.

This analysis suggests that the risk factors for the diagnosis of ML are age >46 years, LDH >173 U/l, as well as sIL2R >1,946 U/ml. However, the diagnostic value of higher age [Sn 0.17, Sp 0.287, PPV 0.598, NPV 0.750, accuracy 0.625, LR+ 1.286, negative likelihood ratio (LR-) 0.288] and LDH (Sn 0.767, Sp 0.452, PPV 0.617, NPV 0.627, accuracy 0.621, LR+ 1.400, LR- 0.515) was relatively small compared with that of higher levels of sIL2R (Sn 0.346, Sp 0.930, PPV 0.852, NPV 0.552, accuracy 0.617, LR+ 4.972, LR- 0.703). When these factors were combined with sIL2R, the diagnostic value mildly improved (Sn 0.293, Sp 0.957, PPV 0.886, NPV 0.539, accuracy 0.601, LR+ 6.744, LR- 0.739) compared with that of sIL2R alone (Table V).

In summary, sIL2R appears to be a more useful predictive marker compared with any other clinical parameters for the diagnosis of lymphomas.

## Discussion

The present study demonstrated the diagnostic characteristics of sIL2R in the diagnosis of lymphomas. There have been few reports evaluating the value of serum sIL2R levels for the differential diagnosis of lymphoma from other conditions in the clinical setting.

Nakase *et al* (7) reported that the sIL2R levels are elevated in patients with hematological neoplasms and non-hematological solid tumors compared with those in healthy subjects. Furthermore, extremely high levels (>3,000 U/ml) are only observed in acute leukemia or malignant lymphomas. These findings suggest that sIL2R levels may be a useful marker for the differential diagnosis between hematological neoplasms and non-hematological solid tumors in patients with bulky diseases (7). Patients with infectious diseases were excluded from this study. Similar results were observed in pediatric patients with leukemia, lymphoma and malignant solid tumors (3). As lymphoma may mimic a wide variety of diseases, the differential diagnosis of infectious or inflammatory diseases is crucial in patients presenting with inflammatory symptoms.

Tsujioka *et al* retrospectively analyzed sIL2R levels in consecutive ML patients and compared them with those in non-hematological diseases categorized by initial diagnosis (8). They compared the sIL2R levels between patients with ML and patients in the control group, which was divided into 6 categories as follows: Autoimmune diseases, non-hematological tumors, infections, fever of unknown origin (FUO), lymphadenopathy, and others. They observed that sIL2R was moderately increased in non-hematological diseases, with higher levels at 1,500 U/ml, at which level the PPV and LR+ were also elevated, indicating diagnostic accuracy for lymphomas.

In the present study, control groups were categorized by final diagnosis according to the observed clinical outcome. Our data also suggest that the diagnostic threshold of sIL2R, in which ML is considered, is at 1,104-1,500 U/ml, at which point Sp is elevated to 80%. In our cohort, the optimal cutoff level of sIL2R for the diagnosis of lymphoma was 1,946-2,000 U/ml, at which point the Sn was 35% and the Sp was 93%, strongly suggesting the diagnosis of ML. At those levels, serum sIL2R appears to be more useful compared with any other non-invasive marker for the diagnosis of lymphomas.

In the present study, aggressive lymphomas exhibited the highest serum sIL2R levels; however, indolent lymphomas exhibited a mild elevation, similar to other disease categories. Furthermore, among aggressive lymphomas, the sIL2R levels in patients with T/NK lymphomas (even when ATLL was excluded) were significantly higher compared with those in patients with aggressive B-cell lymphomas. These findings are consistent with previous reports (6–8).

In Japan, serum sIL2R measurement has been introduced in clinical practice (1,4,6–8,10–12) and has been commercially available and covered by insurance since October 1994, for the purpose of evaluating the response after treatment and disease monitoring after remission in patients with ATLL and non-Hodgkin lymphomas. Furthermore, from April 2006 onwards, sIL2R has been utilized for screening as a tumor marker when there is suspected ATLL or non-Hodgkin lymphoma. At present, sIL2R is often used for patients with suspected lymphomas for screenings and differential diagnosis of tumors with uncommon presentation, lymphadenopathy with atypical course, cases with FUO, or even unidentified complaints, as lymphoma may present with various clinical symptoms, mimicking a number of conditions (13), and may be considered in the differential diagnosis of a wide variety of diseases.

It appears difficult to distinguish ML from other conditions. However, the present study reported some evocative findings. Extremely high sIL2R levels strongly suggest the diagnosis of aggressive lymphoma, although common false-positive exceptions should be considered, including advanced non-lymphoid solid tumors with splenic involvement, drug allergies presenting with marked inflammatory symptoms, and tuberculosis or other infectious diseases. In addition, some common false-negative cases include indolent lymphomas or patients with low tumor burden.

We herein attempted to evaluate sIL2R as a tumor marker for screening and differential diagnosis using data collected from consecutive patients with suspected lymphoma who had their sIL2R levels measured in various clinical presentations. Further investigations are required to evaluate the usefulness of sIL2R in specific clinical settings to clearly determine the subcategory of patients with clinical symptoms or characteristics for whom sIL2R measurement may prove useful. For example, sIL2R was found to be useful as a diagnostic marker in hemophagocytic syndromes/hemophagocytic lymphohistiocytosis associated with lymphomas (14–16).

In conclusion, as serum sIL2R levels may be elevated in several pathological conditions, including inflammatory or neoplastic diseases, they appear to be a less specific marker. However, the findings of the present study indicate that higher levels of sIL2R strongly suggest the presence of lymphoma, and may thus be useful for the diagnosis of aggressive types of lymphomas, after ruling out other false-positive conditions.

## Figures and Tables

**Table I.** Patient characteristics.

Diagnosis	n	M/F	Mean age (range), years	Fever (+/-)
Lymphomas	120	71/49	64.1 (21–90)	16/104
Aggressive	97	62/35	64.6 (21–90)	15/82
Indolent	23	9/14	61.8 (32–82)	1/22
Other lymphoid neoplasms				
Plasma cell disorders	8	4/4	62.3 (48–70)	0/8
CLL	5	1/4	70.4 (54–83)	0/5
Non-hematolymphoid tumors	50	27/23	65.4 (19–87)	5/45
Histologically proven	43	22/21	65.0 (19–87)	5/38
Not histologically proven	7	5/2	68.0 (49–79)	0/7
Infection	32	12/20	43.0 (20–78)	14/18
Lymphadenopathy	23	9/14	38.0 (20–76)	10/13
Gastrointestinal	7	2/5	55.0 (27–78)	2/5
Others	2	1/1	63.0 (56–70)	2/0
Non-infectious inflammation	18	11/7	54.1 (24–80)	5/13
Autoimmune	10	6/4	61.8 (28–80)	2/8
Allergy	3	1/2	47.7 (24–71)	2/1
Others	5	4/1	60.6 (28–80)	1/4
Miscellaneous	15	7/8	65.1 (35–85)	1/14

CLL, chronic lymphocytic leukemia; M, male; F, female.

**Table II.** Histological diagnosis of patients with lymphoid neoplasms.

Category	Diagnosis	M/F	Mean age (range), years
Aggressive lymphomas	Diffuse large B-cell lymphoma	41/27	66 (24–90)
	Intravascular large B-cell lymphoma	1/2	66 (69–76)
	Mantle cell lymphoma	4/1	72 (46–83)
	Follicular lymphoma, grade 3A	3/1	62 (54–81)
	Classical Hodgkin's lymphomas	4/0	46 (21–59)
	Peripheral T-cell lymphoma	2/0	57 (50–64)
	Angioimmunoblastic T-cell lymphoma	3/1	77 (71–79)
	ATLL	0/1	60
	NK/T-cell lymphoma	4/0	49 (26–71)
	Methotrexate-related LPD	0/2	58 (55–61)
Indolent lymphomas	Follicular lymphoma, grade 1–2	3/5	62 (43–77)
	MALT type lymphoma	3/9	63 (32–82)
	Nodal marginal zone B-cell lymphoma	1/0	32
	Lymphoplasmacytic lymphoma	1/0	62
	Cutaneous T-cell lymphoma	1/0	68
CLL	B-CLL	1/4	70 (54–83)
Plasma cell disorders	Multiple myeloma	1/1	64 (59–68)
	Plasmacytoma	1/0	56
	POEMS syndrome^a^	0/1	48
	AL-amyloidosis	1/0	70
	Light chain deposition disease	0/1	70
	IgM-MGUS	1/1	63.5 (51–76)

a Polyneuropathy, organomegaly, endocrinopathy, monoclonal gammopathy and skin changes. NK, natural killer; LPD, lymphoproliferative disease; MALT, mucosa-associated lymphoid tissue; CLL, chronic lymphocytic leukemia; MGUS, monoclonal gammopathy of undetermined significance; ATLL, adult T-cell lymphoma/leukemia; M, male; F, female.

**Table III.** Comparison of characteristics between the ML and other groups.

	ML group		Other group		
			
	Median (range)	Mean ± SD	Median (range)	Mean ± SD	P-value
Age, years	67 (21–90)	64.2±13.3	60 (19–87)	57.4±18.9	0.02
WBC count (cells/*µ*l)	4,570 (1,950–84,590)	8,136±9,745	4,680 (1,920–17,470)	6,753±3,008	0.89
C-reactive protein (mg/dl)	0.40 (0–17.3)	2.12±3.59	0.20 (0–27.1)	1.99±4.40	0.07
Lactate dehydrogenase (U/l)	217 (97–1,269)	295±219	182 (99–4435)	308±565	<0.01
sIL2R (U/ml)	920 (159–58,089)	3,240±7,312	520 (150–4,433)	786±727	<0.01

ML, malignant lymphoma; SD, standard deviation; WBC, white blood cell; sIL2R, serum soluble interleukin-2 receptor.

**Table IV.** Diagnostic characteristic in each cut-off level of sIL2R for the diagnosis of ML.

Cut-off sIL2R level (U/ml)	ML/other	Sn	Sp	PPV	NPV	Accuracy	LR+	LR-	OR
≥500	105/61	0.79	0.47	0.63	0.66	0.64	1.4	0.45	3.32
<500	28/54								
≥1,000	63/27	0.47	0.77	0.70	0.56	0.61	2.02	0.69	2.93
<1,000	70/88								
≥1,500	52/15	0.39	0.87	0.78	0.55	0.61	3.00	0.70	4.28
<1,500	81/100								
≥2,000	46/8	0.35	0.93	0.85	0.55	0.62	4.97	0.70	7.07
<2,000	87/107								
≥2,500	37/4	0.28	0.97	0.90	0.54	0.60	8.00	0.75	10.7
<2,500	96/111								
≥3,000	35/3	0.26	0.97	0.92	0.53	0.59	10.1	0.76	13.3
<3,000	98/112								
≥4,000	26/1	0.20	0.99	0.96	0.52	0.57	22.5	0.81	27.7
<4,000	107/114								
≥5,000	20/0	0.15	1.00	1.00	0.50	0.54	Inf	0.85	–
<5,000	113/115								

ML, malignant lymphoma; Sn, sensitivity; Sp, specificity; PPV, positive predictive value; NPV, negative predictive value; LR+, positive likelihood ratio; LR-, negative likelihood ratio; OR, unadjusted odds ratio; Inf, infinite (could not be evaluated).

**Table V.** Diagnostic characteristic in each risk group for the diagnosis of ML.

Risk factors	ML/other	Sn	Sp	PPV	NPV	Accuracy	LR+	LR-
Age, years								
>46	122/82	0.92	0.29	0.60	0.75	0.63	1.29	0.29
≤46	11/33							
LDH, U/l								
>173	102/63	0.77	0.45	0.62	0.63	0.62	1.40	0.52
≤173	31/52							
sIL2R, U/ml								
>1,946	46/8	0.35	0.93	0.85	0.55	0.62	4.97	0.70
≤1,946	87/107							
Number of risk factors								
0	5/18	0.04	0.84	0.22	0.43	0.41	0.24	1.14
	128/97							
1	25/46	0.19	0.60	0.35	0.39	0.38	0.47	1.35
	108/69							
2	64/46	0.48	0.60	0.58	0.50	0.54	1.20	0.87
	69/69							
3	39/5	0.29	0.96	0.89	0.54	0.60	6.74	0.74
	94/110							

ML, malignant lymphoma; Sn, sensitivity; Sp, specificity; PPV, positive predictive value; NPV, negative predictive value; LR+, positive likelihood ratio; LR-, negative likelihood ratio.

## Data Availability

The datasets used and/or analyzed during the present study are available from the corresponding author on reasonable request.

## Abbreviations

ALL: acute lymphoblastic leukemia
ATLL: adult T-cell lymphoma/leukemia
AUC: area under the curve
CLL: chronic lymphocytic leukemia
CRP: C-reactive protein
FUO: fever of unknown origin
IL-2: interleukin 2
LDH: lactate dehydrogenase
LR+: positive likelihood ratio
LR-: negative likelihood ratio
NPV: negative predictive value
PPV: positive predictive value
sIL2R: serum soluble interleukin-2 receptor
Sn: sensitivity
Sp: specificity
ROC: receiver operating characteristic
WBC: white blood cell
